# Subjective well-being among PhD students in mainland China: the roles of psychological capital and academic engagement

**DOI:** 10.3389/fpsyg.2024.1354451

**Published:** 2024-01-18

**Authors:** Fei Cao, Li-fang Zhang, Mengting Li, Zhengli Xie

**Affiliations:** ^1^Faculty of Education, The University of Hong Kong Special Administrative Region, Hong Kong, China; ^2^School of Education Science, Nanjing Normal University, Nanjing, China; ^3^Faculty of Humanities, The Hong Kong Polytechnic University Special Administrative Region, Hong Kong, China

**Keywords:** subjective well-being, PhD students, psychological capital, academic engagement, mainland China

## Abstract

The mental well-being of PhD students is a major concern in higher education. However, very few studies have investigated the influencing factors of PhD students’ subjective well-being (SWB) – an important indicator of mental well-being. Even no study on the influencing factors of PhD students’ SWB has been undertaken in mainland China. Based on job demands–resources theory, the present study pioneers the investigation of the relationship between PhD students’ psychological capital (PsyCap; comprising self-efficacy, hope, resilience, and optimism) and SWB (comprising positive affect, negative affect, and life satisfaction) in mainland China. It further examined the mediating role of academic engagement (comprising vigor, dedication, and absorption) in this relationship. PhD students (*n* = 376) from two comprehensive universities in Jiangsu province responded to an online survey. The results showed that (1) self-efficacy was positively associated with life satisfaction, hope was positively associated with positive affect, optimism was significantly associated with all three dimensions of SWB, but resilience was not significantly associated with any of the three SWB dimensions; and (2) dedication mediated the relationship between hope and life satisfaction and that between optimism and negative affect and life satisfaction, but vigor and absorption did not mediate any of the PsyCap–SWB relationships. Limitations and practical implications of this study are discussed.

## Introduction

There has been tremendous growth in research on PhD education amid the massification of PhD education in recent years (McAlpine et al., 2020). One increasingly popular stream of research has moved beyond studying the common themes in PhD education (e.g., student attrition, supervision, and degree completion) (McAlpine et al., 2020) to paying attention to PhD students’ mental well-being. A global survey published in *Nature* reported high rates of mental health problems among PhD students (Woolston, 2019), and PhD students in China fare the worst (for example, only 55% of the Chinese students who responded to the survey said that they were at least partially satisfied with their PhD experience, while the satisfaction rate was 72% among the respondents outside China) due to such factors as heavy workloads and a lack of support (Woolston and O’Meara, 2019). The COVID-19 pandemic has made PhD students further isolated, uncertain, and academically struggling (Sverdlik et al., 2022). Poor mental health is a serious issue in itself (Kotera et al., 2021) and has further repercussions for PhD students, including low academic productivity (Wollast et al., 2023) and high dropout rates (Phan, 2023). Nonetheless, the factors affecting PhD students’ mental well-being in a Chinese context remain understudied.

Among various influencing factors, this study particularly focused on personal resources that is defined as individuals’ beliefs in their ability to control and successfully influence their environment (Hobfoll et al., 2003). According to job demands–resources (JD–R) theory—a prominent theory of well-being, personal resources is an essential element for promoting individual well-being (Demerouti et al., 2001; Bakker and Demerouti, 2017). Furthermore, there is an underlying mechanism in the relationship between personal resources and well-being (named the motivational process); specifically, personal resources bolster well-being through enhanced engagement (Demerouti et al., 2001; Bakker and Demerouti, 2017). Based on JD-R theory, the present study investigated the relationship between an important type of psychological resources – PsyCap (i.e., a positive psychological state of development), as proposed by Luthans (2002) – and PhD students’ subjective well-being (SWB) in mainland China, and further examined the mediating role of academic engagement in this relationship.

This study enriches the literature on PhD education in general and, more specifically, on PsyCap, academic engagement, and SWB. For practice, this study has implications for stakeholders in PhD education (including higher education institutions, supervisors, PhD students, and their family members) in their efforts to enhance PhD students’ SWB.

## Mental well-being and PhD students

Mental well-being research consists of two dominant orientations: hedonia and eudaimonia. Hedonia is described as an experience, while eudaimonia is a way of positive functioning (Huta and Waterman, 2014). The most widely used conceptualization of hedonia is Diener et al.’s (2002) tripartite model of subjective well-being (SWB). SWB, colloquially referred to as *happiness*, concerns individuals’ evaluations of their lives as a whole (Diener et al., 2002). High levels of SWB are indexed by frequent positive affect, infrequent negative affect, and high life satisfaction (Diener et al., 2002). These three SWB dimensions are related but independent of one another (Diener and Emmons, 1984; Lucas et al., 1996). One of the most common theories of eudaimonia is Ryff’s, 1989 model of psychological well-being. Psychological well-being encompasses autonomy, environmental mastery, personal growth, positive relations, purpose in life, and self-acceptance. Previous studies on PhD students’ mental well-being mostly focused on the hedonia orientation (e.g., Pychyl and Little, 1998; Zahniser et al., 2017; Moate et al., 2019; Sverdlik et al., 2022) and very few studies took the eudaimonia perspective (e.g., Devine and Hunter, 2017; F Lynch et al., 2018).

Considering PhD students’ conditions, the present study stressed SWB as the indicator of PhD students’ mental well-being. During their PhD journey, students undergo an emotionally intensive process (Corcelles et al., 2019) and experience above-average stress (Barry et al., 2018) which is detrimental to their general well-being. Positive and negative affect can reflect PhD students’ emotional states and life satisfaction is a robust predictor of health and general well-being (Xie et al., 2023). Moreover, research has also shown that SWB is positively related to self-rated health among Chinese emerging adults (Liu et al., 2023).

Despite the abundant literature on SWB, only two empirical studies have examined the factors influencing PhD students’ SWB based on the three commonly investigated dimensions: Moate et al. (2019) investigated the effect of perfectionism on PhD students’ SWB in Canada, and Pychyl and Little (1998) explored the influence of the Big Five personality traits and personal projects on PhD students’ SWB in the United States.

There are three main limitations in the existing studies. First, the findings of the abovementioned studies conducted in Western cultures may be difficult to apply to non-Western cultural settings such as China. Cultures can differ in how strongly the components of SWB relate to each other, the mean level of SWB, and the correlates of SWB with certain factors covarying more strongly in one cultural context versus another (Tov and Nai, 2018). Therefore, it is necessary to investigate PhD students’ SWB in a non-Western context.

Second, neither of the abovementioned studies investigated the role of psychological resources in PhD students’ SWB. According to JD–R theory (Demerouti et al., 2001; Bakker and Demerouti, 2017), personal resources are important in promoting well-being. The present study pioneers the investigation of the role of PsyCap in PhD students’ SWB.

Third, academic engagement, which refers to a positive, fulfilling, learning-related state of mind (Schaufeli et al., 2002a, 2002b), is considered the most important factor in student development in higher education (Hu and Kuh, 2002); however, no study has examined its role in PhD students’ SWB. JD–R theory (Demerouti et al., 2001; Bakker and Demerouti, 2017) states that personal resources can promote well-being through engagement. To better understand the mechanisms underlying the relationship between PhD students’ PsyCap and their SWB, this study examines academic engagement as a potential mediator.

### Psychological capital and subjective well-being

JD–R theory posits that personal resources can promote individuals’ well-being (Demerouti et al., 2001; Bakker and Demerouti, 2017). Personal resources have been frequently conceptualized as PsyCap in the literature (Liu et al., 2020). PsyCap is a composite construct (comprising self-efficacy, hope, resilience, and optimism) that stems from positive psychology (Luthans, 2002). The four-dimensional PsyCap has been extensively supported in the literature (Mikus and Teoh, 2022), and each dimension is theory-based, measurable, open to development, and positively associated with desirable outcomes (Luthans, 2002).

According to Bandura’s (1997) social cognitive theory, self-efficacy refers to individuals’ confidence in their ability to execute a course of action to achieve a desired outcome. Self-efficacy influences emotions (i.e., affect) through cognitive, behavioral, and emotional orientations that enable individuals to select and apply effective emotion regulation strategies (Bandura, 1997). As self-efficacious people are predisposed to perceive a stressor as an opportunity rather than a challenge (Hajek and König, 2019), they are more likely to buffer the negative effects of stress on life satisfaction (Burger and Samuel, 2017).

In the hope theory of Snyder et al. (1991), hope is defined as the willpower that motivates individuals to achieve goals and the waypower to identify multiple routes to goal achievement. With their perceptions of adequate willpower and waypower, high-hope individuals are more likely to perceive goal attainment and experience more positive affect and less negative affect (Snyder et al., 1991). Meanwhile, because hopeful people strive to achieve their goals and can generate alternative solutions to do so, they tend to be more satisfied with their achievements in life (Kwok et al., 2015).

According to Masten (2001), resilience is the capability of recovering from adversity and adapting to stressful events. Resilient people can use diverse coping strategies (e.g., relaxation techniques and humor) that can not only cultivate positive affect and mitigate negative affect (Tugade and Fredrickson, 2004) but also improve life satisfaction (Wang et al., 2019).

As presented by Scheier and Carver (1985), optimism is a general expectation that positive outcomes will occur in the future. As this expectation leads to behaviors that bring optimists closer to their goals (Lucas et al., 1996), optimists are expected to obtain more positive outcomes and have greater life satisfaction than pessimists. Furthermore, because optimists can use adaptive coping responses to manage stressful events (Nes and Segerstrom, 2006), they are expected to be able to regulate their emotions through adaptive coping strategies.

Numerous studies have shown the favorable effects of PsyCap on SWB (e.g., Krok, 2015; Hajek and König, 2019; Labrague, 2021). For example, in England, PhD students’ self-efficacy promoted their positive affect and reduced their negative affect (Jackman and Sisson, 2021). In a US sample, hope positively predicted life satisfaction (Pleeging et al., 2020). However, only one study examined the contributions of all four PsyCap dimensions to all three SWB dimensions. Specifically, Denovan and Macaskill (2017) found that among undergraduates in the UK, self-efficacy and optimism positively predicted life satisfaction and positive affect but negatively predicted negative affect, and that hope and resilience did not predict any SWB dimension. These results indicate that the PsyCap dimensions have different effects on the SWB dimensions. Hence, this study examines the relationship between the four dimensions of PsyCap and the three dimensions of SWB among PhD students, which can enable a better understanding of the two constructs. The first hypothesis of this study is as follows:

*H_1_*: Self-efficacy, hope, resilience, and optimism would be positively related to positive affect and life satisfaction and negatively related to negative affect among PhD students.

### Academic engagement as a mediator

As mentioned earlier, JD–R theory postulates a motivational process in which personal resources contribute to better well-being via engagement (Demerouti et al., 2001; Bakker and Demerouti, 2017). Academic engagement refers to a positive, fulfilling, learning-related state of mind that is characterized by vigor (i.e., high levels of energy and mental resilience while studying, willingness to make an effort to study, and persistence when facing obstacles), dedication (i.e., a sense of significance, enthusiasm, inspiration, pride, and challenge in academic tasks), and absorption (i.e., a pleasant state of full immersion in study, in which time passes quickly and it is difficult to detach oneself from studying) (Schaufeli et al., 2002a,b). PsyCap promotes academic engagement by strengthening students’ belief in their ability to adequately complete their academic tasks and achieve the desired results (Sonnentag et al., 2010). Engaged students are likely to have a high level of SWB because they regard their studies as interesting, pleasurable, and satisfying (Caesens et al., 2014).

Although the relationships between the four PsyCap dimensions and the three academic engagement dimensions have not been empirically tested, some indications can be gleaned from studies revealing the positive effect of PsyCap on work engagement (Garrosa et al., 2011; Ouweneel et al., 2012; Nishi et al., 2016; Zuo et al., 2021). For example, entrepreneurs’ self-efficacy positively predicted their vigor, dedication, and absorption in work (Zuo et al., 2021). Likewise, no study to date has examined the associations between the three academic engagement dimensions and the three SWB dimensions. Prior research has found that composite work engagement mediated the relationship between composite PsyCap and employees’ positive and negative affect (Adil and Kamal, 2016) and life satisfaction (Karatepe and Karadas, 2015). In addition, Datu and King (2018) found that composite academic engagement positively predicted life satisfaction and positive affect but negatively predicted negative affect. Nonetheless, no study has investigated how each of the three academic engagement dimensions mediates the relationships between the four PsyCap dimensions and the three SWB dimensions. The present study makes such an attempt to reveal the detailed relationships between these key research variables. Accordingly, the second hypothesis is as follows:

*H_2_:* Vigor, dedication, and absorption would mediate the relationship of self-efficacy, hope, resilience, and optimism to positive affect, negative affect, and life satisfaction among PhD students.

## Methods

### Sample and procedure

Prior to data collection, ethics approval had been obtained from the Human Research Ethics Committee of The University of Hong Kong. The data for this study were collected through an online questionnaire sent by staff members to PhD students in all fields of study enrolled at two comprehensive universities in Jiangsu province, mainland China between December 2021 and January 2022. Responses were received from 511 participants who gave their informed consent by clicking the link to start the survey, representing a response rate of approximately 57%. The removal of invalid returned questionnaires was based on one or more of three reasons: (1) overly patterned responses (i.e., consistently selecting the same answer on all items of at least one dimension); (2) unusually short response time (less than the minimum time required to read all questions based on pilot testing); or (3) extreme outliers that were identified as values deviating more than three times the interquartile range from the upper or lower quartile (Hartmann and Opaas, 2023) using SPSS) 27. The final sample consisted of 376 participants aged 21–45 years (*Mean*_age_ = 27.39 years, *SD_age_* = 3.17, *Median*_age_ = 27 years). Detailed information on the research sample can be found in Table 1.

**TABLE 1:** Sample characteristics.

Variable	Category	Number (%)
Gender	Male	181 (48.1)
	Female	195 (51.9)
Institutional selectivity	‘Double first-class’ university	197 (52.4)
	Non-‘double first-class’ university	179 (47.6)
Program year	Year 1	141 (37.5)
	Year 2	125 (33.2)
	Year 3	75 (19.9)
	Above year 3	35 (9.3)
Academic discipline	Hard-pure	85 (22.6)
	Hard-applied	123 (32.7)
	Soft-pure	95 (25.3)
	Soft-applied	73 (19.4)
Annual family income	Less than US$11,508	115 (30.6)
	US$11,508–21,578	158 (42.0)
	US$21,578–71,927	90 (23.9)
	More than US$71,927	13 (3.5)
Prior research experience	Yes	312 (83.0)
	No	64 (17.0)
Marital status	Unmarried	298 (79.3)
	Married	78 (20.7)
	Divorced	0 (0.0)
	Widowed	0 (0.0)

The ‘Double first-class’ initiative was issued by the Chinese government that aimed at building a growing number of world-class universities and disciplines by the year 2030.

### Measures

*PsyCap* was measured by the Chinese version (Liu et al., 2020) of the Psychological Capital Questionnaire (PCQ; Luthans et al., 2007), which has been adapted for use among Chinese PhD students for assessing PsyCap in academic domains. The PCQ contains 24 items, with six items assessing each of four subscales: self-efficacy, hope, resilience, and optimism. Sample items are provided in Table 2. The participants were asked to rate the items on a 7-point Likert scale, with 1 indicating *strongly disagree* and 7 indicating *strongly agree* with the statements describing how they think about themselves.

**TABLE 2:** Sample items in the four inventories.

Inventory	Scale	Sample item
Psychological Capital Questionnaire	Self-efficacy	I feel confident analysing a long-term problem to find a solution.
	Hope	At this time, I am meeting the study goals that I have set for myself.
	Resilience	I usually manage difficulties one way or another in my research.
	Optimism	I approach my research as if ‘every cloud has a silver lining’.
Utrecht Work Engagement Scale-Student	Vigor	As far as my studies are concerned, I always persevere, even when things do not go well.
	Dedication	I am enthusiastic about my studies.
	Absorption	Time flies when I am studying.
Positive and Negative Affect Schedule	Positive affect	Excited.
	Negative affect	Guilty.
Satisfaction with Life Scale.	Life satisfaction	I am satisfied with my life.

*Academic engagement* was assessed through the Chinese version (Fang et al., 2008) of the Utrecht Work Engagement Scale–Student (UWES–S; Schaufeli et al., 2002a,b). The UWES–S is a 17-item scale with three subscales: vigor (six items), dedication (five items), and absorption (six items). The participants rated themselves on a 7-point Likert scale, with 1 indicating *never* and 7 indicating *always* regarding the learning states described in the statements (see also Table 2 for sample items).

*SWB* was evaluated by the Chinese version (Ye, 2008) of the Positive and Negative Affect Schedule (PANAS; Watson et al., 1988) and the Satisfaction with Life Scale (SWLS; Diener et al., 1985). The PANAS contains 10 items assessing positive affect and 10 items assessing negative affect. The SWLS is a five-item measure of life satisfaction. The response format for the PANAS and SWLS was a 7-point Likert scale, with 1 indicating *strongly disagree* and 7 indicating *strongly agree* with the statements (see also Table 2 for sample items).

### Data analysis

There was no missing value in the data. An inspection of the skewness and kurtosis coefficients for each of the dimensions of the key variables proved normal (skewness coefficients ranged from −1.12 to 0.38 and kurtosis coefficients from −0.55 to 1.59). The psychometric properties of the three main inventories were determined, including construct validity evaluated by confirmatory factor analysis (CFA) in M*plus* 7.4 and internal consistency calculated by Cronbach’s alpha in SPSS 27. Descriptive statistics were then examined.

To test the research hypotheses more precisely, the PhD students’ demographics were considered. Research has shown that PsyCap differs as a function of institutional selectivity (Elliott, 2016) and prior research experience (Hadley et al., 2007), and SWB differs as a function of gender (Diener et al., 1999), marital status (Diener et al., 2000), and family income (Pleeging et al., 2020). Research has also shown significant differences in academic engagement by gender (Luo, 2017). The multivariate analysis of variance results indicated that the three main research variables differed by each of these demographics. Therefore, partial correlations between the three main research variables were analyzed, controlling for demographic characteristics.

To test *H*_1_, a hierarchical multiple regression analysis was performed in SPSS 27. To test *H*_2_, a path analysis using the maximum likelihood estimator was carried out in M*plus* 7.4, with the means of the respective dimension of each research variable used as the observed variables. A bias-corrected bootstrap 95% confidence interval (CI) procedure (Hayes, 2017) with 5,000 samples was implemented to test for indirect effects. The indirect effects were considered significant when the 95% CI did not include zero (Preacher and Hayes, 2008). The model fit was evaluated using the chi-square test of exact fit, comparative fit index (CFI), Tucker–Lewis index (TLI), root mean square error of approximation (RMSEA), and standardized root mean squared residual (SRMR). The following recommended cut-off points were taken to indicate an adequate model fit: χ^2^*/df* < 5, CFI and TLI values >0.90, and RMSEA and SRMR values <0.08 (Hu and Bentler, 1999).

## Results

### Descriptive statistics

CFA was conducted to test the fit of the measurement model. We compared the following models: (1) the four-factor PsyCap (containing four latent constructs and 24 observed indicators that were specified to load only on their respective latent constructs) with a one-factor PsyCap (all 24 indicators were loaded together on one latent construct), (2) the three-factor academic engagement (containing three latent constructs and 17 observed indicators that were specified to load only on their respective latent constructs) with a one-factor UWES–S (all 17 indicators were loaded together on one latent construct), and (3) the three-factor SWB (containing three latent constructs and 25 observed indicators that were specified to load only on their respective latent constructs) with a one-factor SWB (all 25 indicators were loaded together on one latent construct).

The results (see Table 3) demonstrated that the multi-factor models (i.e., the four-factor PsyCap, three-factor academic engagement, and three-factor SWB) fit the data better than their respective one-factor models. The revised multi-factor models of PsyCap and academic engagement and the original multi-factor model of SWB showed a good fit to the sample data (see Table 3), confirming the good construct validity of each inventory.

**TABLE 3:** Measurement model.

	χ^2^	*df*	*p*	CFI	TLI	RMSEA	SRMR	Model comparison	Δχ^2^	Δ*df*
*Psychological Capital*										
M_1_ (Four-factor psychological capital)	884.60	246	< 0.001	0.87	0.86	0.08	0.06			
M_2_ (One-factor psychological capital)	1,431.33	252	< 0.001	0.76	0.74	0.11	0.07	M_2_ vs. M_1_	546.73***	6
M_3_ (Revised M_1_)	536.35	230	< 0.001	0.94	0.93	0.06	0.05			
*Academic engagement*										
M_4_ (Three-factor academic engagement)	690.18	116	< 0.001	0.86	0.84	0.12	0.06			
M_5_ (One-factor academic engagement)	921.90	119	< 0.001	0.80	0.78	0.13	0.06	M_5_ vs. M_4_	231.72***	3
M_6_ (Revised M_4_)	237.49	102	< 0.001	0.97	0.96	0.06	0.04			
*Subjective Well-being*										
M_7_ (Three-factor subjective well-being)	844.07	272	< 0.001	0.91	0.90	0.08	0.05			
M_8_ (One-factor subjective well-being)	2625.69	275	< 0.001	0.61	0.57	0.15	0.11	M_8_ vs. M_7_	1,781.62***	3

M3 and M6 were revised based on modification indices. Specifically, in M3, 16 residual variance correlations (i.e., item 18 with items 15 and 17, item 11 with item 9, item 4 with items 1 and 3, item 21 with item 20, item 14 with item 13, item 23 with item 19, item 17 with items 24 and 14, item 18 with item 8, item 3 with item 1, item 24 with items 6, 9, 11, and 12) were added to the model. In M6, 14 residual variance correlations (i.e., item 11 with item 10, item 8 with item 6, item 16 with items 14 and 15, item 17 with item 12, item 14 with items 13, 15, and 16, item 12 with items 3, 5, and 9, item 2 with item 1, item 12 with item 3, item 13 with item 17) were added to the model.

***p < 0.001.

Once the measurement model was established, the descriptive statistics were examined. Table 4 displays the means, standard deviations, partial correlations, and reliability coefficients of the study variables. Cronbach’s alpha coefficients were all above the 0.70 criterion (Hair et al., 2010). As expected, partial correlation coefficients indicated that all dimensions of key variables were positively related to one another, except for negative affect that was negatively related to all the other dimensions of key variables. Given that some of the partial correlation coefficients were high (above 0.70), the variance inflation factors (VIF) for each of the predictors were tested for multicollinearity. The maximum VIF values were below the recommended value of 5 (Kutner et al., 2004), indicating that multicollinearity was not an issue.

**TABLE 4:** Descriptive statistics, partial correlations, and internal consistency.

Variable	Mean	*SD*	1	2	3	4	5	6	7	8	9	10
1. Self-efficacy	5.51	0.78	*0.84*									
2. Hope	5.35	0.83	0.61	*0.84*								
3. Resilience	5.38	0.82	0.56	0.72	*0.8*2							
4. Optimism	5.55	0.87	0.54	0.68	0.67	*0.87*						
5. Vigor	5.64	0.82	0.26	0.41	0.37	0.36	*0.80*					
6. Dedication	5.68	0.98	0.43	0.53	0.44	0.55	0.73	*0.89*				
7. Absorption	5.49	0.98	0.30	0.44	0.38	0.39	0.70	0.72	*0.88*			
8. Positive affect	5.08	0.91	0.44	0.56	0.47	0.56	0.53	0.71	0.57	*0.91*		
9. Negative affect	2.92	1.19	−0.28	−0.28	−0.28	−0.33	−0.29	−0.38	−0.28	−0.49	*0.81*	
10. Life satisfaction	5.23	1.07	0.38	0.39	0.33	0.50	0.29	0.46	0.35	0.58	−0.43	*0.87*

Demographics (i.e., gender, institutional selectivity, marital status, family income, and prior research experience) were controlled for in the partial correlations. Cronbach’s alpha values are presented in the diagonals.

All partial correlations were significant at *p* < 0.001.

### The relationship between psychological capital and subjective well-being

After controlling for gender, institutional selectivity, marital status, family income, and prior research experience, PsyCap uniquely accounted for 34% of the variance in positive affect, 13% in negative affect, and 24% in life satisfaction. As shown in Table 5, self-efficacy positively predicted life satisfaction but did not significantly predict positive or negative affect. Hope positively predicted positive affect but did not significantly predict negative affect or life satisfaction. Optimism positively predicted positive affect and life satisfaction and negatively predicted negative affect. Resilience did not significantly predict any of the SWB dimensions.

**TABLE 5:** Regression results of the relationship between psychological capital and subjective well-being.

Criteria	Subjective well-being		
	Positive affect	Negative affect	Life satisfaction
β_self-efficacy_	0.10	−0.12	0.14*
β_hope_	0.29***	−0.03	0.08
β_resilience_	−0.00	−0.05	−0.09
β_optimism_	0.31***	−0.22**	0.42***
*R*^2^_total_	0.43	0.17	0.37
*R*^2^_demographics_	0.09	0.04	0.13
*R*^2^_PsyCap_	0.34	0.13	0.24
*F*	30.77***	8.06***	17.41***
*df*	9, 366	9, 366	9, 366

*p < 0.05, **p < 0.01, ***p < 0.001.

### The mediating role of academic engagement in the relationship between psychological capital and subjective well-being

**FIGURE 1:**
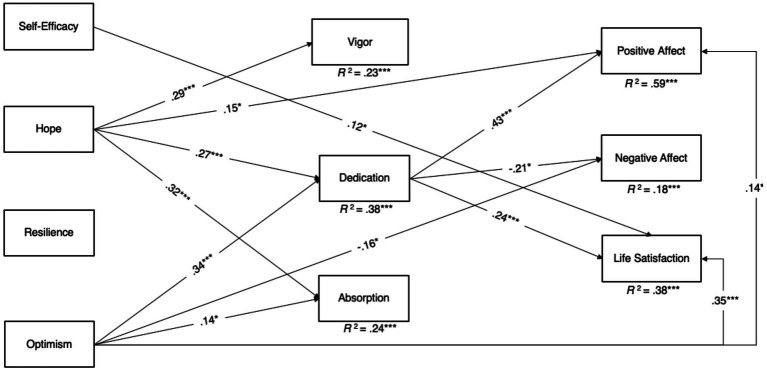
Results of path analysis. For better legibility, nonsignificant paths between the key research variables and paths from demographics t0 the key research variables are not depicted. *p < 0.05, **p < 0.01, ***p < 0.001.

For the purpose of model parsimony, only the significant paths between the control variables and key research variables were retained. The model fit indices indicated that the mediation model (see Figure 1) fit the data very well: χ^2^ = 55.10, *df* = 37, RMSEA = 0.04, CFI = 0.99, TLI = 0.97, and SRMR = 0.04. Standardized estimates of path coefficients, standard deviations, and 95% CIs were estimated. As expected, positive affect (*R*^2^ = 0.59) was positively predicted by hope, optimism, and dedication. Negative affect (*R*^2^ = 0.18) was negatively predicted by optimism and dedication. Life satisfaction (*R*^2^ = 0.30) was positively predicted by self-efficacy, dedication, and optimism beyond gender, marital status, and family income. Vigor (*R*^2^ = 0.21) was positively predicted by hope. Dedication (*R*^2^ = 0.37) was positively predicted by hope and optimism. Absorption (*R*^2^ = 0.24) was positively predicted by hope and optimism. Self-efficacy and resilience were positively predicted by institutional selectivity and prior research experience, whereas negatively predicted by gender. Hope and optimism were positively predicted by institutional selectivity and prior research experience. However, the other direct effects were not significant.

The bias-corrected bootstrapping 5,000 results showed that all of the significant indirect effects involved dedication as a mediator. Specifically, dedication mediated (1) the positive relationship between hope and life satisfaction (estimate = 0.06, SE = 0.03, 95% CI = [0.01, 0.12]); (2) the negative relationship between optimism and negative affect (estimate = −0.07, SE = 0.03, 95% CI = [−0.13, −0.01]); and (3) the positive relationship between optimism and life satisfaction (estimate = 0.08, SE = 0.03, 95% CI = [0.02, 0.14]). However, dedication did not mediate the relationship between hope and optimism with positive affect and that between hope and negative affect. Vigor and absorption did not serve as mediators between any PsyCap dimension and any SWB dimension.

## Discussion

The present study pioneers the investigation of the relationship between PsyCap and PhD students’ SWB and the mediating role of academic engagement in this relationship. The results partially confirmed the hypotheses, and the main findings are discussed below.

### Psychological capital and subjective well-being

The results showed that PsyCap made significant contributions to SWB. First, as expected, PhD students’ self-efficacy was positively related to their life satisfaction. This finding not only dovetails with that of Zeng et al. (2022) but can also be explained. PhD students who perceive a sense of control over their lives, high self-recognition, and greater confidence in their academic abilities should have more life satisfaction than their counterparts (Tamannaeifar and Motaghedifard, 2014). The findings supported *H_1_* based on JD**–**R theory (Demerouti et al., 2001; Bakker and Demerouti, 2017).

However, the finding that self-efficacy was not statistically related to positive and negative affect is inconsistent with prior studies (Tamannaeifar and Motaghedifard, 2014; Hajek and König, 2019; Caprara et al., 2022) and may be explained by the type of self-efficacy measured. Self-efficacy can be generic or domain-specific. Whereas this study measured PhD students’ academic self-efficacy (i.e., one type of domain-specific self-efficacy), prior studies have assessed generic self-efficacy and self-efficacy in expressing positive affect (i.e., another type of domain-specific self-efficacy) (Tamannaeifar and Motaghedifard, 2014; Hajek and König, 2019; Caprara et al., 2022).

Second, as hypothesized, hopeful PhD students had more positive affect. It is possible that hopeful students can deal with difficulties more effectively and persevere when facing failures; thus, they are more likely to achieve valued outcomes and experience more positive affect (Rego et al., 2012). However, there were two findings that are not in line with *H_1_*: PhD students’ hope was not statistically related to their negative affect or life satisfaction. The differential relationship of hope to positive and negative affect found in this study echoes a general finding in the literature that hope is more strongly related to positive affect than to negative affect (Pleeging et al., 2021). The nonsignificant relationship between hope and life satisfaction, despite contradicting that obtained in prior studies (Karataş et al., 2021; Zeng et al., 2022), can be explained. Hope relates to goal-directed behavior (Snyder et al., 1991). Given that within the intensely competitive research environment (Arimoto, 2009), PhD students’ goal-directed behavior may not necessarily ensure they achieve desired goals (e.g., high-quality publications), some hopeful PhD students may not be satisfied with life.

Third, PhD students’ resilience was not statistically related to any aspect of SWB, which concords with Denovan and Macaskill (2017). Unlike the proactive nature of the other PsyCap dimensions, resilience is more reactive because the capacity to bounce back and move beyond can only be displayed when external events (e.g., stressful events) occur (Luthans et al., 2007). The present finding might thus be attributed to the participants in this study tending to deal with stress proactively rather than reactively. This aligns with Confucianist philosophies that have a different interpretation of adversity (i.e., viewing adversity as opportunities to develop for self-growth) from a typical Western understanding (i.e., perceiving adversity as obstacles) (Ni et al., 2014).

Fourth, optimism was the only PsyCap dimension to have predictive utility on all three SWB dimensions, which is congruent with prior studies (Krok, 2015; Hajek and König, 2019) and supports *H_1_* based on JD**–**R theory (Demerouti et al., 2001; Bakker and Demerouti, 2017). This finding indicates that optimism is the most important factor in affecting PhD students’ SWB, possibly because optimistic students can use adaptive coping strategies (Nes and Segerstrom, 2006) to regulate their emotions and they are more likely to achieve goals (Lucas et al., 1996), which may lead to greater life satisfaction.

### Academic engagement as a mediator

The results showed that dedication mediated the relationship between hope and life satisfaction and the relationship of optimism to negative affect and life satisfaction. These findings suggest that students with willpower and waypower (i.e., who are hopeful) are more dedicated in learning (e.g., have more enthusiasm and inspiration) and have more life satisfaction, and that students who expect positive results (i.e., who are optimistic) are more dedicated in learning and experience negative affect (e.g., worry and anxiety) less frequently and life satisfaction more frequently than their counterparts. These findings echo those of studies that have found that work engagement mediates the association of PsyCap with life satisfaction (Karatepe and Karadas, 2015) and negative affect (Adil and Kamal, 2016) and support JD–R theory’s assumption on personal resources’ motivational process (Demerouti et al., 2001; Bakker and Demerouti, 2017).

However, dedication did not mediate the relationship of self-efficacy and resilience to SWB. The nonsignificant relationship of self-efficacy and resilience to dedication was also found by Wang et al. (2017). One plausible interpretation is that although PhD students have confidence in their academic abilities, they may not have a sense of significance and pride (i.e., dedication) in learning because it is not easy to produce significant research. Meanwhile, as mentioned above, PhD students who participated in this study might not rely on reactive psychological resources (i.e., resilience) for being academically engaged.

Vigor and absorption did not show any significant mediation effect. Especially, no significant relationship was found between vigor and absorption and any SWB dimension. Although vigor and dedication are commonly considered core aspects of engagement (Sweetman and Luthans, 2010), the present results indicated that only dedication was important for PhD students’ SWB. This suggests that PhD students who believe that their research is meaningful and are proud of their research feel happier than other students. In summary, the above findings partially supported *H_2_* based on JD–R theory (Demerouti et al., 2001; Bakker and Demerouti, 2017).

## Practical implications and limitations

### Practical implications

The general findings that PsyCap (specifically self-efficacy, hope, and optimism) and academic engagement (specifically dedication) plays important roles in PhD students’ SWB have practical value for higher education institutions, supervisors, PhD students, and their family members.

Knowledge of the significant roles of self-efficacy, hope, and optimism in PhD students’ SWB can help higher education institutions to organize PsyCap intervention programs (especially those aimed at enhancing self-efficacy, hope, and optimism) for PhD students to improve their SWB. Artificial intelligence-assisted psychosis risk screening methods (e.g., chatbot; Cao and Liu, 2022) can also be used to enhance PhD students’ self-efficacy, hope, and optimism. Supervisors can improve students’ SWB by providing constructive and positive feedback to improve students’ self-efficacy, assisting students in setting goals and generating pathways when facing difficulties to promote hope, and helping students analyze what is within and out of their control and figuring out how to take actions when facing setbacks to enhance optimism (Luthans et al., 2006). PhD students can enhance their SWB by strengthening their self-efficacy through mastery experiences and vicarious learning (Bandura, 1997), setting SMART (Specific, Measurable, Achievable, Relevant, and Time-bound) goals to enhance hope, and using strategies, such as self-talk about positive and realistic expectations and ‘best positive self’ exercises, to maintain their optimism (Salanova and Ortega-Maldonado, 2019).

With the understanding that dedication played a significant role in PhD students’ SWB, supervisors and students’ family members could identify ways of promoting students’ dedication in learning in their efforts to improve students’ SWB. For instance, supervisors can guide PhD students to design meaningful and challenging projects that give them a sense of significance, pride, and challenge in academic tasks. Supervisors should also steer PhD students in the right direction and provide flexibility to match students’ enthusiasm and inspiration. Additionally, because dedicated PhD students need time and energy for academic tasks, students’ family members may take on more household duties on the students’ behalf (Aryee et al., 1999) to enhance students’ dedication.

### Limitations

This study has five main limitations. First, the cross-sectional design impedes drawing causal conclusions. Future research could use a longitudinal design to elucidate the temporal pattern of the research variables and to address causality.

Second, the data were collected by the student self-reported method. Although this method is commonly used for data collection, it may result in common method bias. Future research could collect data using multiple approaches (e.g., different response formats and data sources) to improve the reliability of the findings.

Third, the study was carried out with participants from two comprehensive universities in mainland China. Thus, the findings may not be generalized to other cultures, countries, or even institutions for three reasons: 1) there exist cultural differences as mentioned above; 2) PhD students in China report the highest rates of mental health problems (Woolston and O’Meara, 2019); and 3) PhD programs vary across institutions and countries (Pappa et al., 2020). Additionally, voluntary participation could be prone to self-selection bias. For instance, PhD students who were more conscious of their mental health might have been more likely to respond to the questionnaire. Thus, future research should be conducted in other contexts using random sampling to validate our findings.

Fourth, the key research variables in this study only included PhD students’ psychological factors. Environmental factors, such as social support, may also influence PhD students’ SWB. We plan to explore the influence of environmental factors on PhD students’ SWB more broadly in future studies.

Finally, due to space limit, demographics served only as control variables in this study. It would be meaningful in future studies to explore how the hypothesized relationships in this study vary with PhD students’ demographics.

## Data availability statement

The raw data supporting the conclusions of this article will be made available by the authors, without undue reservation.

## Ethics statement

The studies involving human participants were reviewed and approved by the University of Hong Kong’s Human Research Ethics Committee (HREC). The studies were conducted in accordance with the local legislation and institutional requirements. The participants provided their written informed consent to participate in this study.

## Author contributions

FC: Conceptualization, Data curation, Formal analysis, Investigation, Methodology, Validation, Writing - original draft, Writing - review & editing. L-fZ: Conceptualization, Investigation, Methodology, Supervision, Validation, Writing - review & editing. ML: Methodology, Writing - review & editing. ZX: Investigation, Writing - review & editing.
